# Highly efficient base editing in bacteria using a Cas9-cytidine deaminase fusion

**DOI:** 10.1038/s42003-018-0035-5

**Published:** 2018-04-19

**Authors:** Ke Zheng, Yang Wang, Na Li, Fang-Fang Jiang, Chang-Xian Wu, Fang Liu, Huan-Chun Chen, Zheng-Fei Liu

**Affiliations:** State Key Laboratory of Agricultural Microbiology and Key laboratory of Preventive Veterinary Medicine in Hubei Province, College of Veterinary Medicine, Huazhong Agricultural University, Wuhan, 430070 China

## Abstract

The ability to precisely edit individual bases of bacterial genomes would accelerate the investigation of the function of genes. Here we utilized a nickase Cas9-cytidine deaminase fusion protein to direct the conversion of cytosine to thymine within prokaryotic cells, resulting in high mutagenesis frequencies in *Escherichia coli* and *Brucella melitensis*. Our study suggests that CRISPR/Cas9-guided base-editing is a viable alternative approach to generate mutant bacterial strains.

## Abstract (web summary)

Ke Zheng and colleagues repurposed a nickase Cas9-cytidine deaminase fusion protein to effectively direct the conversion of cytosine to thymine on bacterial genome. This study suggests that CRISPR/Cas9-guided base-editing can be used to generate viable mutant bacterial strains.

## Introduction

Programmable modification of genomes is a vital approach to the study of gene function^1–3^. Recombineering using the recA and Lambda Red systems are prevalent methods for constructing modified strains in various bacteria species^4–10^. RecA-mediated recombination needs long homologous target gene sequences (≥500 bp), and occurs at a low frequency (10^−6^ to 10^−4^)^4,5^. In contrast, the λ Red system shows high recombination efficiency (~10^−3^ to 10^−1^), and requires as little as 30 bp of homologous sequence to serve as a substrate^6–10^. Recently, the CRISPR (clustered, regularly interspaced, short palindromic repeats) /Cas9 system^1,2^ was employed to assist editing of bacterial genomes by killing non-edited cells, leading to a recovery of almost 100% edited cells^11,12^. However, donor DNA as editing template is still required to knock out genes in bacterial cells using these methods.

Recently, a system called “Base Editor” (abbreviated to BE) was reported, which fused cytidine deaminase to a CRISPR-associated Cas9 variant to convert cytosine (C) to uracil (U) at targeted sites without double-strand breaks (DSBs), resulting in C → T (or G → A) substitution^13^. The base-editing system directly edits single nucleotides, which avoids the dependence on homology-dependent repair (HDR). Various derivations of the BE system, such as BE1, BE2 and BE3, have been developed and all the derivations induce base-editing in mammalian cells with very low indel rates. These systems have been applied to mammalian cells, animals and plants^13–18^. Other CRISPR-guided BE systems using human AID (activation-induced cytidine deaminase)^19^ or AID ortholog PmCDA1 from sea lamprey^20^ have been reported and demonstrated in eukaryotic organisms^21,22^. Some researchers used BE3 to engineer premature coding termination to provide an alternative approach to knock out genes^15,18,23^. We envisioned that a highly efficient BE3 system would enable rapid and efficient programmable editing in bacteria.

In the present work, we constructed bacterial expressing BE3 plasmids that induced the substitution of C to T to convert a Gln codon (CAA) to a stop codon (TAA) in the *tetA* gene conferring tetracycline resistance at close to 100% efficiency in *E*. *coli* strain XL1-Blue (tetracycline insensitive). Next, the *lacZ* and *rppH* genes were selected to further validate the same method in *E*. *coli*. We also demonstrated that BE3 can achieve precise and efficient base conversion within *Brucella melitensis* (*B. melitensis*) in a targeted manner. Collectively, the BE3 editor was shown to be a powerful tool for direct base changes in *E. coli* and *B. melitensis*.

## Results

### *E. coli* genome editing using BE3

**Fig. 1:**
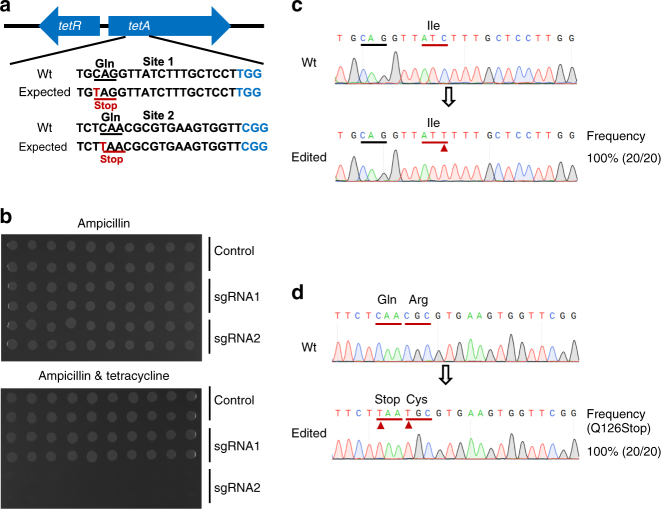
Induction of base conversion at *tetA* sites in *E. coli*. **a** The designed mutation sites in the *tetA* gene. PAM motif (blue), target sites (red). **b** Colony tetracycline-resistance test. **c** Sanger sequencing at *tetA* gene site 1. **d** Sanger sequencing at *tetA* gene site 2. The substituted bases are marked with a red arrow. The transformations and base-editing assays were repeated for three times. The phenotype assay and Sanger sequencing were performed on 20 colonies of each group. The editing frequency was calculated by formula (edited colony/total colony)

First, we tested whether BE3 can function in *E. coli* cells. The BE3 editing element was cloned into an inducible expression vector, under control of the PL_lacO-1_ promoter (Supplementary Fig. 1, Supplementary Note 1). The *E. coli* strain XL1-Blue was selected as a model because the Tn*10* transposon containing the tetracycline-resistance gene *tetA* is located in its genome^24^. We predicted that the CAG/CAA (Gln) or CGA (Arg) codons would be converted into respective TAG/TAA/TGA premature Stop codons, thereby inactivating the TetA protein tetracycline efflux pump function. The sgRNAs were designed within the first third of the *tetA* open reading frame to truncate the protein (Fig. 1a). The base-editing occurred during the transformation recovery step when the BE3 protein was expressed and performed its function (1 h post heat shock). After base-editing, the edited *E. coli* cells were isolated by plate streaking to avoid satellite colony contamination and then grown in liquid cultures. A 2 μL volume of each liquid culture was dropped on plates (≥2.605 × 10^7^ colony-forming unit (CFU), concentration: 1.3025 × 10^10^ CFU mL^−1^, Supplementary Table 1, Supplementary Table 2) to detect the loss of antibiotic resistance (Fig. 1b). For the BE3-induced incapacitating TetA at site 2, all colonies (20/20) showed a loss of function (Fig. 1b). Mutagenesis at both sites was confirmed by PCR amplification and Sanger sequencing. For the sgRNA1-guided mutagenesis, the base substitution occurred at an undesired codon (ATC|Ile to ATT|Ile) rather than at the designed one (Fig. 1c). The sequencing of tetracycline-sensitive colonies showed that the CAA|Gln codon was converted to a TAA|Stop codon, and that other codons were also changed by base conversion (Fig. 1d). Editing was 100% efficient at some sites.

### Editing efficiency of BE3 in *E. coli*

**Fig. 2:**
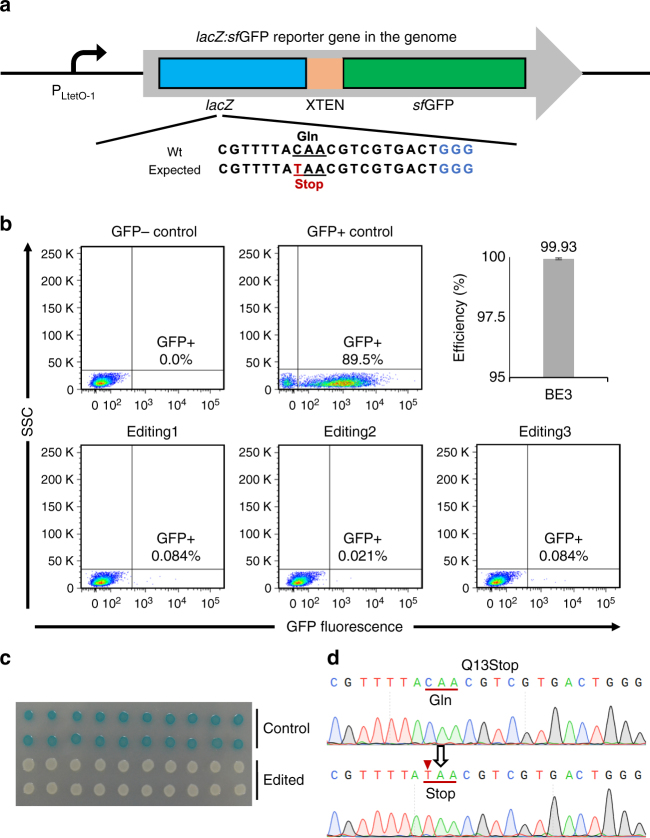
Highly efficient base-editing in *E. coli*. **a** Schematic representation of the *lacZ*:*sf*GFP reporter gene in engineered *E. coli* strain GS1783-*lacZ*:*sf*GFP. **b** Flow cytometry analysis of base-editing efficiency based on GFP fluorescence. The morphological complexity of cells was measured by side scatter light (SSC). Three biological replicates were assayed. *E. coli* strain GS1783 was used as a GFP-negative control and *E. coli* strain GS1783-*lacZ*:*sf*GFP was used as a GFP-positive (GFP^+^) control. The editing efficiency was calculated by formula: editing frequency = $1 - \frac{{{\mathrm{Percentage}}\;{\mathrm{of}}\;{\mathrm{GFP}}^ + {\mathrm{cells}}\;{\mathrm{in}}\;{\mathrm{edited}}\;{\mathrm{population}}}}{{{\mathrm{Percentage}}\;{\mathrm{of}}\;{\mathrm{GFP}}^ + {\mathrm{cells}}\;{\mathrm{in}}\;{\mathrm{unedited}}\;{\mathrm{population}}}}$. **c** Resulting phenotypes (X-gal reaction indicated LacZ activity) of isolated *E. coli*. **d** Sanger sequencing of *lacZ*:*sf*GFP gene editing. The substituted base is marked with a red arrow. The transformations and phenotype assays were repeated for three times. The phenotype assay and Sanger sequencing were performed for 20 colonies of each group. The editing frequency was calculated by formula (edited colony/total colony)

**Fig. 3:**
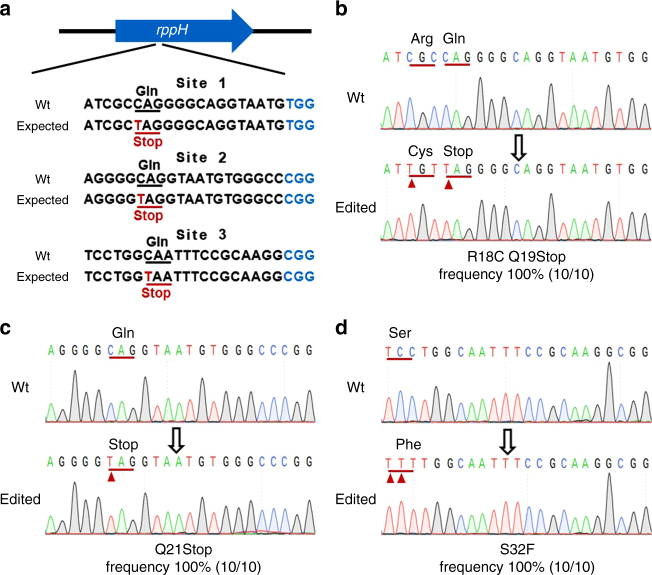
Induction of base conversion at *rppH* sites in *E. coli*. **a** The designed mutation sites in the *rppH* gene. PAM motif (blue), target sites (red). **b** **c**, **d** Sanger sequencing of base-editing at different loci in the *rppH* gene. The substituted bases are marked with red arrows. The transformations and base-editing assays were repeated for three times. The phenotype assay and Sanger sequencing were performed on 10 colonies of each group. The editing frequency was calculated by formula (edited colony/total colony)

Next, we constructed a *lac*Z:*sf*GFP reporter gene-integrated *E. coli* strain to calculate the accuracy of editing efficiency. A DNA fragment containing the P_LtetO-1_ promoter, which controlled the *lacZ*:sfGFP-fused reporter gene and the chloramphenicol-resistance gene (Fig. 2a), was PCR amplified from pXG-10sf^25^. This fragment harbored homologous sequence and integrated into the genome through λ red-mediated recombination. The base-edited colonies were cultured in LB medium and analyzed by flow cytometry and X-gal cytochemistry. Flow cytometry showed that 99.93% of edited *E. coli* cells lost fluorescence (Fig. 2b), indicating that the BE3-mediated base-editing in *E. coli* was almost 100% efficient. X-gal reactions and Sanger sequencing confirmed the editing (Fig. 2c, d). We also tested BE3 on the *rppH* gene and produced highly efficient editing (Fig. 3). In addition, cytotoxicity when using BE3 was measured by transformation. Compared to wtCas9, BE3 protein is non-lethal when it targets and nicks the genome (Supplementary Fig. 2).

### *B. melitensis* genome engineering using BE3

**Fig. 4:**
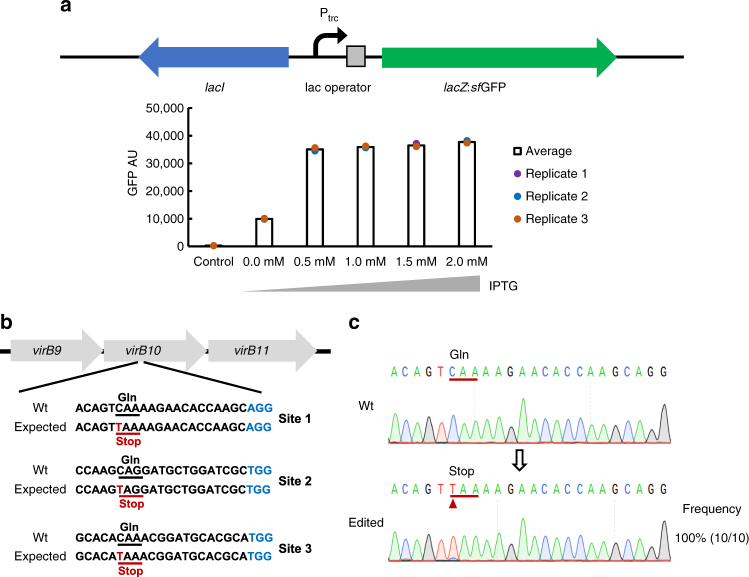
Premature stop codon generated by the BE3 system in *B. melitensis*. **a** Expression levels of *lacZ*:*sf*GFP from the pZK79-*lacZ*:*sf*GFP vector under repressed and induced conditions. **b** The designed mutation sites in the *virB10* gene. PAM motif (blue), target sites (red). **c** Sanger sequencing at *virB1*0 gene site 1. The substituted base is marked with a red arrow. The transformations and base-editing assays were repeated for three times. The phenotype assay and Sanger sequencing were performed on 10 colonies of each group. The editing frequency was calculated by formula (edited colony/total colony)

We next applied this editor in *B. melitensis*, an α proteobacteria and facultative intracellular bacterial pathogen. To apply the base-editing system in *B. melitensis*, we constructed an IPTG-inducible expression vector based on the broad host-range plasmid, pBBR1-MCS5^26^, and tested inducing conditions (Fig. 4a). The BE3 gene was cloned into the inducible expression vector under control of the trc promoter (Supplementary Fig. 3, Supplementary Note 2). Three sgRNAs were designed for mutagenesis of the *virB10* gene (Fig. 4b). The transformants were cultured in TSB medium, followed by inactivation and genomic DNA extraction. The target regions were amplified and sequenced. The conversion occurred at only one site among the candidates with 100% efficiency (Fig. 4c).

We demonstrated that CRISPR/Cas9-guide-specific base conversion could be achieved in Gram-negative bacteria, *E. coli* and *B. melitensis*, with high efficiency. The conversion at expected sites resulted in premature termination of coding genes, which abolished protein function. This finding simplifies the process of base mutagenesis and expands the applications of the CRISPR/Cas9 system in bacterial cells.

## Discussion

Recently, programmable base conversion has been reported in *E. coli* by fusing cytidine deaminase with ZF (zinc finger) or TALE (transcription activator-like effectors)-DNA binding domains^27^. A single copy GFP reporter gene locus possessing a ‘broken’ start codon (‘ACG’) was rescued by deamination with up to 13% efficiency in an uracil repair gene-deleted *E. coli* strain. In our study, the CRISPR/Cas9-guided BE3 system achieved much higher efficiency because a single-strand DNA ‘R loop’ structure^28^, a natural substrate for APOBEC1 deaminase^29^, is generated by Cas9 unwinding activity^28^. The break on the non-edited strand mediated by nCas9 enhances the trend towards the expected editing^13^.

Some limitations remain for using base-editing as a way to knock out a gene: first, the premature stop codon depends on CAA/CAG/CGA/TGG codons; second, a truncation site should be at an appropriate location in an ORF; additionally, we should not ignore the fact that the editing efficiency of the BE systems followed the order TC ≥ CC ≥ AC ≥ GC^13^.

Recently, improved BE systems have been reported. On the basis of Cas9 homology and engineered variants, improved base editors with different protospacer adjacent motif (PAM) specificities were developed and demonstrated in mammalian cells^30^ and zebrafish^31^. Furthermore, the width of the deamination window can be narrowed to 1–2 nt by engineered APOBEC1 enzymes^30^. Notably, programmable base editing of A to G was reported recently^32^, which broadens the application of base editing. The wild-type *E. coli* tRNA-specific adenosine deaminase (ecTadA)-dCas9 fused protein with no ability of deaminate adenine at target loci in DNA was transformed into high efficient adenine base editors (ABEs) after a series of evolution and engineering. The ABEs show high efficient A-to-G editing in both *E. coli* and human cells. The BEs and ABEs enable programmable editing of all four nucleotide without DSBs. Taken together, we show that CRISPR-guided BE3 can perform highly efficient base-editing in bacterial cells, which may reduce the time and efforts that take to manipulate bacterial genes and to obtain viable mutant bacterial strains.

## Methods

### Construction of pEcBE3 and pBmBE3 vectors

The original plasmid containing the BE3 element (rAPOBEC1-XTEN-Cas9n-UGI-NLS) was obtained from Addgene (pCMV-BE3, #73021)^13^. The *Bsa*I restriction sites in the BE3 element were removed through site-directed mutagenesis by Gibson assembly. The mutated BE3 was cloned into the *Pml* I site in the inducible expression vector pZF17–31 (Supplementary Fig. 3, Supplementary Note 2) by Gibson assembly, resulting in pZF17–32 (Supplementary Fig. 4, Supplementary Note 3). The sgRNA gene was amplified from pZF17–33 (Supplementary Fig. 5, Supplementary Note 4) and cloned into the *Xho*I site in pZF17–32, resulting in inducible expression vector pEcBE3 (Supplementary Fig. 1, Supplementary Note 1) for use in base editing in *E. coli*. The pBmCRISPR2 vector (Supplementary Fig. 6, Supplementary Note 5) used for expressing Cas9 and sgRNA in *B. melitensis* was used as the vector backbone for pBmBE3. The Cas9 coding sequence was replaced by the mutated BE3 element through Gibson assembly, to obtain pBmBE3 (Supplementary Fig. 7, Supplementary Note 6). For sgRNA cloning, synthesized oligonucleotides were annealed to form a dimer, which was then ligated into *Bsa*I-digested pEcBE3 or pBmBE3. PCR was performed using Phanta Max Super-Fidelity DNA Polymerase (Vazyme), and Gibson assembly was performed according to a reported protocol^33^. Oligonucleotide sequences are listed in Supplementary Table 3–4.

### Base-editing assay and screening

We performed the base-editing assay during the transformation recovery step. Chemically competent *E. coli* cells were transformed with pEcBE3 series plasmids. After heat-shock, transformed *E. coli* cells were incubated in SOC medium (containing 0.6 mM IPTG) at 37 °C shaking at 180 r.p.m for 1 h. Cells were then spread on LB agar plates (containing 50 μg mL^−1^ ampicillin and 0.6 mM IPTG). For mutagenesis in *B. melitensis*, pBmBE3 series plasmids were transformed into strain 16 M electro-competent cells by electroporation. The transformed cells were recovered in TSB (Tryptic Soy Broth, BD) medium (containing 0.6 mM IPTG) at 37 °C shaking at 180 r.p.m. for 4 h and were then spread on TSA plates (containing 50 μg mL^−1^ gentamycin and 0.6 mM IPTG). Empty BE3 vectors carrying non-targeting sgRNA were transformed as control. Single colonies were then cultured in LB medium (for *E. coli*) or TSB medium (for *B. melitensis*) and genomic DNA extracted using a bacterial genome DNA extraction kit (TIANGEN). Since satellite colonies occurs when the ampicillin-resistance gene contained in pEcBE3 series plasmids is used, it is necessary to isolate the transformed *E. coli* colonies so as to avoid contamination being contaminated by satellite colonies. For each plate, the colonies were resuspended in 1 mL of LB medium, and spreaded on LB agar plate (containing 50 μg mL^−1^ ampicillin and 0.6 mM IPTG) using sterile incubation loop according to T-Streak methods. The plate then was incubated at 37 °C overnight to obtain single colony. The target regions were amplified by PCR and sequenced to confirm mutagenesis. The mineral oil was added to PCR tube to avoid aerosol contamination. The primers used are listed in Supplementary Table 3.

### *E. coli* reporter strain construct

The *lacZ*:*sf*GFP gene was selected as a reporter gene. The *lacZ*:*sf*GFP-fused gene and chloramphenicol-resistance gene (Cm^R^) were amplified from pXG-10sf^25^ using two cycle PCR, which harbored homologous sequence targeting the *E. coli* strain GS1783^34^ genome. GS1783 cells were prepared as electro-competent cells according to a previous study^34^. The gel-purified PCR product was electro-transformed into GS1783 cells. After incubation in 1 mL SOC medium at 37 °C with shaking at 180 r.p.m., the transformed cells were screened on LB agar plates containing 34 μg mL^−1^ chloramphenicol. The primers used are listed in Supplementary Table 3.

### Inducing conditions of the trc promoter in *B. melitensis*

The *lacZ*:*sf*GFP gene was cloned into the *Nsi*I and *Xba*I sites of the inducible expression vector, pZK79 (Supplementary Fig. 8, Supplementary Note 7, in previous study not reported), to obtain pZK79-*lacZ*:*sf*GFP. The pZK79 and pZK79-*lacZ*:*sf*GFP were electro-transformed into *B. melitensis* 16 M competent cells, respectively. Transformants were cultured in 5 mL TSB medium (50 μg mL^−1^ gentamycin) for 48 h, and then in fresh 5 mL TSB medium (50 μg mL^−1^ gentamycin) at a ratio of 1:50 until the OD_600_ reached ~0.6. IPTG was then added to gradient final concentration (0 mM, 0.5 mM, 1.0 mM, 1.5 mM and 2.0 mM) and the culture continued for 6 h. Phenol was then added at a final concentration of 1% (v/v) to inactivate the culture. Cells were then collected and washed with PBS (phosphate-buffered saline buffer). GFP fluorescence was measured using a PerkinElmer EnVision plate reader (the OD_600_ value was used to normalize cell concentration). Primers used are listed in Supplementary Table 3.

### Phenotype assay

Isolated single colonies were cultured in LB medium (50 μg mL^−1^ ampicillin) and 2 μL of bacterial culture then spread on LB agar plates. In the LacZ activity assay, we used ampicillin (50 μg mL^−1^) and X-gal (40 μg mL^−1^) LB agar plates. In the tetracycline-sensitive assay, we used ampicillin (50 μg mL^−1^) or ampicillin (50 μg mL^−1^) plus tetracycline (10 μg mL^−1^) LB agar plates. Bacterial culture concentration was assessed by optical density (OD_600_) measurement and serial dilution-plate count. We measured the OD_600_ value of 20 bacterial cultures (tetA-editing), and selected the culture with the lowest value for the serial dilution-plate count.

### Flow cytometry assay

Transformed GS1783-LacZ:sfGFP cells were resuspended in 2 mL PBS buffer, and diluted 100-fold. The diluted cells were analyzed using a BD FACSVerse flow cytometer. The GS1783 cells and untransformed GS1783-LacZ:sfGFP cells were taken as GFP-negative and GFP-positive control cells, respectively.

### Cytotoxicity assay of BE3

To compare cytotoxicity of wtCas9 and BE3 on *E. coli* cells, the plasmid pZK77 (Supplementary Fig. 9, Supplementary Note 8) expressing wtCas9 and sgRNA was constructed based on pwtCas9-bacteria (Addgene: #44250) by inserting a synthesized sgRNA gene into the *Avr*II site. The pZK77::RppH site was constructed using the same method as for pEcBE3::RppH. DH5α chemically competent cells (Shanghai Weidi Biotechnology) were transformed with 100 ng of pEcBE3::RppH, pEcBE3, pZK77::RppH or pZK77. The empty non-targeted pEcBE3 and pZK77 were controls. After heat-shock, cells were recovered in 1 mL SOC medium for 1 h at 37 °C with shaking at 180 r.p.m. The transformed cells were diluted 10^3^-10^5^-fold or not diluted (for pZK77::RppH) and plated onto Amp LB agar plates.

### Statistical analysis

The *t* test was used to analyze the difference between experiments.

### Data availability

All the data generated or analyzed during this study are included in this published article (and its supplementary information files).

## Electronic supplementary material

Supplementary Information
